# Health Benefits of Endurance Training: Implications of the Brain-Derived Neurotrophic Factor—A Systematic Review

**DOI:** 10.1155/2019/5413067

**Published:** 2019-06-24

**Authors:** Włodzimierz Mrówczyński

**Affiliations:** Department of Neurobiology, Chair of Biological Sciences, Poznan University of Physical Education, 27/39 Królowej Jadwigi St., 61-871 Poznań, Poland

## Abstract

This article presents a concept that wide expression of brain-derived neurotrophic factor (BDNF) and its receptors (TrkB) in the nervous tissue, evoked by regular endurance training (ET), can cause numerous motor and metabolic adaptations, which are beneficial for human health. The relationships between the training-evoked increase of endogenous BDNF and molecular and/or physiological adaptations in the nervous structures controlling both motor performance and homeostasis of the whole organism have been presented. Due to a very wide range of plastic changes that ET has exerted on various systems of the body, the improvement of motor skills and counteraction of the development of civilization diseases resulting from the posttraining increase of BDNF/TrkB levels have been discussed, as important for people, who undertake ET. Thus, this report presents the influence of endurance exercises on the (1) transformation of motoneuron properties, which are a final element of the motor pathways, (2) reduction of motor deficits evoked by Parkinson disease, and (3) prevention of the metabolic syndrome (MetS). This review suggests that the increase of posttraining levels of BDNF and its TrkB receptors causes simultaneous changes in the activity of the spinal cord, the substantia nigra, and the hypothalamic nuclei neurons, which are responsible for the alteration of the functional properties of motoneurons innervating the skeletal muscles, for the enhancement of dopamine release in the brain, and for the modulation of hormone levels involved in regulating the metabolic processes, responsively. Finally, training-evoked increase of the BDNF/TrkB leads to a change in a manner of regulation of skeletal muscles, causes a reduction of motor deficits observed in the Parkinson disease, and lowers weight, glucose level, and blood pressure, which accompany the MetS. Therefore, BDNF seems to be the molecular factor of pleiotropic activity, important in the modulation processes, underlying adaptations, which result from ET.

## 1. Introduction

Endurance activity is a natural form of movement based on aerobic metabolism and repeated isotonic contractions of large skeletal muscles [1, 2]. Cycling, running, and swimming performed at low intensities from minutes to hours by at least several weeks are classical examples of such activity [2, 3]. It is commonly known that endurance training (ET), which is a form of organized and planned endurance activity, brings many health benefits by improving or restoring physical condition. Therefore, it is used not only for sport purposes but also for rehabilitation of patients with neuromuscular [4], cardiovascular [5], and metabolic [6] diseases.

The influence of ET on the skeletal muscles is well known. Regular exercises increase both the density of capillaries in muscle fibers and the flow of blood to whole active muscles [7]. Moreover, endurance activity increases maximal oxygen uptake [8] and improves the ability of the skeletal muscles to produce energy through oxidative metabolism [3] due to an increase in the number and size of mitochondria in trained muscles [9]. Endurance intervention enhances the muscles oxidative capacity [10, 11], triggers the muscle to produce more efficient forms of contractile proteins [12], and modifies the motor unit proportions towards more resistance subtypes [13].

However, ET not only evokes adaptive change in the morphological, metabolic, and contractile properties of trained muscles but also exerts numerous effects on tissues and organs located outside of the activated muscles, thus improving physical competence of the whole organism [14]. Regular endurance effort alterates the functional action of spinal motoneurons, which control the activity of the skeletal muscles [15–17], prevents metabolic syndrome (MetS) [18], regulates fat metabolism [19], decreases blood glucose levels [20], delays the onset of type 2 diabetes [21], and finally reduces the risk of cardiovascular diseases and heart complications and improves the cardiac function [1, 22]. Next, endurance activity also counteracts and delays the development of some neurodegenerative diseases [23, 24] and mental disorders [25].

Moreover, ET can influence the activity of hormonal [26] and immune systems [27], upregulate the level of endogenous antioxidant enzymes [28], improve the mechanical properties and mineral density of bones [29], counteract the risk of osteoporosis [30], and delay the aging processes [31].

Benefits of ET for health are so evident that this type of physical activity has been considered as a drug [32], leading to improvements in life quality and reduction of hospital admission risks [33]. Moreover, ET is often recommended as “a cornerstone in the prevention, management, and treatment of numerous chronic conditions” such as obesity, type 2 diabetes, hypertension, or coronary heart disease [34].

Up to date, reports addressing various physiological and metabolic consequences of endurance exercises [35–38], which are accessible in the PubMed databases, count more than 370000 entries. However, the described physiological, biochemical, and molecular mechanisms behind the numerous adaptations resulting from ET come from studies performed on rodents rather than human beings because it is “difficult to use humans to examine exercise training alterations in many molecular systems as well as most organ systems” [39].

Specifically, majority of studies have been performed on rats (*Rattus norvegicus*) or mice (*Mus musculus)*, because these species share many common structural and functional similarities with humans and hence may shed a light on physiological mechanisms behind the observed effects of endurance exercises [40] and create a possibility to compare obtained results to these performed in humans during compatible effort intensities [41]. Experiments performed on rodents undergoing controlled ET [42] enable obtaining many biochemical, toxicological, or genetic details [43] related to the impact of endurance activity on mammalian organisms.

Despite the fact that most of the studies on the effects of ET were carried out on rodents, there are also incontrovertible evidences from human studies, indicating that regular physical activity is essential in the prevention of chronic diseases and premature death. Warburton et al. [44] described that routine physical activity protects from numerous chronic diseases such as diabetes, osteoporosis, hypertension, obesity, and depression, reduces the incidence of breast and colon cancers, and improves psychological well-being. Physical activity is also an important lifestyle factor, which prevents age-related cognitive decline and dementia in humans [45].

Many effects of endurance exercises, which appears in different tissues and organs suggest that there is no single molecular factor triggering such numerous adaptations after endurance activity. However, a growing number of evidences indicates that plastic alteration associated with endurance activity can be initialized at a molecular level by BDNF—one of the main trophic factors in the nervous system of all mammals [46].

Mammalian neurotrophins include several proteins: nerve growth factor (NGF), BDNF, neurotrophin-3 (NT-3), and neurotrophin-4/5 (NT-4,-5) [47, 48], which all have similar chemical structure [49], but act differently, through the two distinct classes of receptors, namely, transmembrane tyrosine kinase receptors (subtype TrkA, TrkB, or TrkC) and the pan-neurotrophin receptor (p75) [50]. It was indicated that each of the neurotrophins binds with specific Trk receptor, while all of the neurotrophins bind with similar affinity to the p75 neurotrophin receptor [51]. Moreover, coexpression of both types of receptors can lead to a formation of a high-affinity binding complex that enhances Trk signaling and evokes trophic effects [52]. Therefore, both types of receptors can be considered as subunits of neurotrophic receptor [52].

All neurotrophins are responsible for the regulation of neuronal survival, plasticity, and growth during early period of mammal development [53]. However, neurotrophic factors play a more widespread role in adults—participating in many plastic events as regulators of synaptogenesis and synaptic plasticity [54], promotors of regeneration [55], and in learning and memory processes [56, 57]. Hence, they are considered not only as important factors influencing neural growth and differentiation during development but also as potent regulators of plasticity and survival of adult neurons and glia [58].

In mammals, both BDNF and its specific transmembrane tyrosine kinase receptors, TrkB or p75, were found mainly in the nervous system [46, 59, 60]. They were numerously represented in the population of the spinal cord neurons contributing to movement control [61–65], in the hippocampus neurons involved in processes of learning and memory [66], in dopaminergic neurons from the substantia nigra [67], and in hypothalamic nuclei neurons, which regulate energy homeostasis, insulin release, and appetite behavior [48, 68].

Moreover, BDNF and its receptors were also identified in various nonneuronal tissues, namely, in the liver, the pancreas, the adipose tissue, the heart, the endocrine system, and the smooth [69, 70] and skeletal muscles, especially in response to physical exercise [47, 71].

Changes in the BDNF concentration were recognized as an important factor in the pathogenesis of numerous neurological, psychiatric, cardiometabolic, ocular, and skin and urinary system diseases as well as chronic pain-associated disorders [72]. It was shown that a low level of BDNF corresponds to numerous lifestyle-related diseases such as MetS [73] and associated disorders like obesity [74], type 2 diabetes [75], heart failure [76], and acute coronary syndrome [77]. Moreover, low levels of BDNF were also related with the development of four main neurodegenerative diseases with selective death of specific neuronal populations [78] such as Parkinson [79], Alzheimer [80–82], and Huntington diseases [52] and amyotrophic lateral sclerosis [83] as well as with neuropsychiatric disorders such as dementia [84], depression [85], schizophrenia [86], and bipolar disorder [87], which cause severe personal suffering and disability [57].

These findings suggested that a low level of BDNF in circulating blood is a good predictor of a metabolic risk, neurodegenerative or mental illness occurrence, resulting from neural maldevelopment and disturbances in neural plasticity [57].

On the other hand, a considerable elevation of the BDNF concentration in the blood serum and plasma was reported as basic result of ET in healthy humans [66, 88–91] and patients with multiple sclerosis [92]. It was also suggested that a magnitude of BDNF increase can correlate with the intensity of performed exercises [93, 94].

Animal studies demonstrated that the accelerated synthesis of BDNF after increased locomotor activity takes place in the majority of the spinal cord neurons [95], both in healthy and injured animals [63, 96, 97]. Also, a wide expression of BDNF and its TrkB receptors in the spinal cord neurons of mammals was demonstrated by Zhou et al. [61], Scarisbrick et al. [62], and Boyce et al. [64], especially after long-time locomotor training.

Furthermore, the increased production of BDNF was found in various areas of the hippocampus, striatum, hypothalamic nuclei, and brain cortex as a result of endurance activity [68, 90, 98, 99].

It is important to notice that according to Rasmussen et al. [90] about 70-80% of the circulating BDNF is produced by the brain. Pan et al. [100] demonstrated that BDNF can be transported across the blood-brain barrier, which suggests that circulating BDNF can be a good biomarker for the measurement of the BDNF level in the brain. In addition, BDNF is also released by the skeletal muscles into the bloodstream during chronic physical activity [96, 101–103], however, rather in nonsignificant amount, acting exclusively in an auto or paracrine manner [104]. Therefore, animal and human studies brought many evidences that physical exercises can “induce increased expression of BDNF and its release in the brain and peripheral tissues” [105].

However, an increase in the peripheral concentration of BDNF appears rather after aerobic exercises [106–108] than other types of motor activity. For example, data suggest no considerable changes in the levels of the BDNF concentration in the plasma of healthy human after resistance training [94, 107, 109]. Moreover, Huang et al. [107] showed that acute and chronic aerobic exercises can significantly elevate the BDNF concentration in human blood and suggested that this increase may be dependent on exercise intensity. Analysis performed by Szuhany et al. [94] also indicated that aerobic exercise “should be considered as a successful strategy for enhancing of BDNF activity” in humans.

Interestingly, majority of adaptive changes described above were found in neuronal and nonneuronal tissues, which contain a high concentration of either BDNF or its TrkB receptors. Therefore, training-induced elevation of the BDNF level in the blood and its TrkB receptors in some nervous structures seems to be responsible for the modulation of biochemical and/or physiological processes, responsible for the initiation of adaptive changes in different tissues and organs.

## 2. Training-Evoked Increase in the BDNF Level Alters the Properties of Spinal Motoneurons

Motoneurons distributed within the spinal cord and brainstem of mammals represent the final component of neuronal circuits controlling movement [110]. The most important task of these neurons is muscle activation [111], which allow to perform many different motor functions.

Motoneurons receive excitatory and inhibitory synaptic inputs from sensory afferents and from pathways of supraspinal origin either directly or via interneurons [112], integrate synaptic inputs, and finally generate trains of action potentials [113], which are transmitted to the striated muscles. The intrinsic properties of motoneurons and the summation of multiple excitatory and inhibitory synaptic inputs [114] are responsible for the determination of unique properties of firing pattern generated by these neurons, which finally elicit muscle contractions.

The electrophysiological properties of motoneurons depend on several factors including the increase or decrease in physical activity [115]. As a result of physical activity, the electrophysiological properties of motoneurons are modified earlier than the mechanical properties of muscles innervated by them. Therefore, motoneurons constitute an important element of the neuromuscular plasticity being the locus of adaptation to training [115], while muscles, no matter how sophisticated in design and composition, act in response to neural commands [116]. So far, changes in properties of rat motoneurons that precede adaptation in muscles were described after ET [15–17], whole body vibration training [117], muscle overload [118], and strength training [119]. It was demonstrated that long-term (12-16 weeks) endurance running on a treadmill and spinning wheel causes many changes in electrophysiological properties of rat's hindlimb motoneurons. Namely, it leads to a decrease in the neuron resting membrane potential, a decrease in the spike threshold, and an increase in the after hyperpolarization amplitude as well as alterations in the rate of firing [15–17].

Linked to that, considerable expression of BDNF and its TrkB receptors in ventral horn neurons of the spinal cord was detected after both short and long period of locomotor activity at moderate and acute intensities in both normal and spinal transected animals [61–65, 89, 95, 96]. Therefore, alteration in electrophysiological properties of trained motoneurons can result from elevation of the endogenous BDNF level, which promotes plasticity within the spinal cord circuits [120]. For example, changes in motoneuron excitability were observed after exogenous application of the BDNF in hindlimb muscles of rats [121]. Also, considerable alteration in the firing properties of neurons in the trapezoid body [122] and in the oculomotor system [123] was observed in experiments with exposition of these neurons to the exogenous BDNF. Results cited above suggest that increasing the BDNF concentration may change the functional properties of motoneurons.

The level of BDNF in motoneurons can be increased by intensive contractions of skeletal muscles during ET [47, 71, 102, 124]. Rind et al. [125] indicated that BDNF can be retrogradely transported from the muscles by axons and reach transynapically motoneurons, which are endowed with appropriate neurotrophin receptors—TrkB [59]. Gardiner [115] proposed a mechanism of motoneuronal adaptation related to activity, which assumes that ET causes elevation of endogenous neurotrophin concentration (NT-4 and BDNF), which can bind to TrkB receptors in motoneurons. Finally, these processes lead to changes in the gene expression of ion channels resulting in acute modulation of ion channel performance and chronic changes in the properties of spinal motoneurons.

In addition to playing a role in alterations in motoneuron membrane and intrinsic properties, neurotrophins are considered also as activity-related modulators of synaptic transmission [126]. Moreover, two different synaptic effects of neurotrophins were recognized: changes in synaptic transmission and plasticity within seconds or minutes (acute effect) and changes in synaptic structures and function within days (long-term effect) [127], which can occur as a result of ET.

BDNF is mainly known as an activity-dependent modulator of neuronal structure and function in the adult brain, which contributes to numerous synaptic plasticity processes including long-term potentiation, long-term depression, dendritic spine density, or learning and memory encoding and storage (72). However, the participation of BDNF in plastic events on synapses between neurons located at the spinal cord level was reported as well. For example, Lu et al. [128] in experiments performed on spinal cord slice cultures exposed to BDNF indicated changes in excitatory synaptic transmission: increase of excitatory synaptic drive to excitatory neurons and decrease of synaptic excitation on inhibitory neurons. Joseph et al. [97] found that motoneurons and other ventral horn cells distributed in the spinal cord of spinalized rats are able to synthesis BDNF and suggested that postsynaptic release of this neurotrophin can contribute to synaptic plasticity. Wang et al. [129] demonstrated an increase of dendritic extension and synaptic density parallel to an increase of the BDNF expression in lumbar motoneurons of spinalized rats in response to treadmill training. Such results suggest that properties of motoneurons can be modified by synaptic influence from dorsal horn interneurons.

To summarize, membrane and functional properties of motoneurons can be modified by training-evoked increase of the BDNF level; however, the mechanism of these changes is not fully recognized. It is not clear whether they are caused by alterations in properties of spinal motoneurons regulating the activity of skeletal muscles, followed by direct expression of specific ion channels in motor cells or in results of postsynaptic excitatory or inhibitory drive from spinal interneurons.

## 3. Training-Evoked Increase in the BDNF Level Reduces Motor Deficits in Parkinson's Disease

Parkinson's disease (PD) is one of the most common neurodegenerative diseases, which affects 1-4% of human population above the age of 60 [130]. PD is caused by progressive death of dopamine-secreting neurons within the substantia nigra of the midbrain [131, 132]. The substantia nigra sends dopaminergic projections to the basal ganglia that control balance and coordination, being responsible for the execution of voluntary movement [133]. In patients, the loss of dopaminergic inputs from the substantia nigra alters the balance of the output from the basal ganglia to the motor cortex causing the occurrence of motor (resting tremor, rigidity, and stooped posture) and nonmotor (gastrointestinal dysfunction, olfaction disability, and musculoskeletal pain) symptoms [132, 134–136]. The mechanism of PD development is not fully explained, but the combination of genetic and environmental factors (pesticide exposure, beta-blockers, alcohol, deficiency of vitamin D, and traumatic brain injury) can increase the risk of this disease [130, 132, 137].

The progress in neurodegenerative processes occurring in dopaminergic neurons is related to increasing amount of intracellular aggregates containing *α*-synuclein [138]. The appearance of this protein in neurons causes numerous dysfunctions either in mitochondria or in calcium metabolism [139], leading to an enhance production of reactive oxygen species, progressively damaging neurons [140–142]. It was demonstrated that elevated levels of *α*-synuclein impairs retrograde axonal transport of BDNF and suppresses BDNF-mediated trophic signaling cascades [143]. Additionally, inhibition of BDNF/TrkB signaling can result from selective interaction of *α*-synuclein with TrkB receptors [144].

Physical activity is at the top of the list of interventions that prevent PD development in humans [137, 145]. It was demonstrated in patients that treadmill exercises are able to improve the performance of balance-related activities [146], reduce gait disturbances [147], and improve gait speed and stride length [148], forward and backward walking [149], and overground walking speed [150]. The endurance exercises on treadmill is not only therapeutic methods for counteracting motor symptoms in PD but also a suitable way for neurotrophic factor upregulation [151].

PD in experimental animals is usually evoked by infusion of 6-OHDA (6-hydroxydopamine) or MPTP (1-methyl-4-phenyl-1,2,3,6-tetrahydropyridine), which are neurotoxic compounds destroying the dopaminergic neurons [152–156]. It was demonstrated by Lau et al. [157] and Koo et al. [158] that ET is efficient in ameliorating motor PD symptoms evoked in experimental animals. Again, it seems reasonable to link these improvements with an increase of endogenous BDNF and glial cell-derived neurotrophic factor (GDNF) in the substantia nigra which was found in experimental animals after long-term endurance exercises [23, 157]. Results obtained by Real et al. [159] further conclude that during physical exercises, the BDNF–TrkB system is involved in improvement of the dopaminergic system and recovery of motor behavior in 6-OHDA-injected rats. da Silva et al. [151] stressed that both BDNF and GDNF are responsible for the enhancement of dopamine release, due to their participation in the regulation of activity of signaling cascades enabling tyrosine hydroxylase gene transcription.

Recently, Katila et al. [160] showed that Metformin (drug often used against MPTP neurotoxicity in experiments on animal PD model) also increases the level of BDNF in the substantia nigra and activates signaling pathways related to cell survival. However, study performed on the PD inflammatory model of rats demonstrated that Metformin can not only fail to protect the nigral dopaminergic system but also even exacerbated its damage [161]. Such contradictory results suggest that Metformin, which helps to control the level of sugar in human blood [162], should be very carefully used in the treatment of diabetes, especially in patients who may suffer from PD [161].

In conclusion, described reports suggest that training-evoked increase in the BDNF and NT-4 levels in the substantia nigra of mammals with PD can be a reason of increase in the dopamine level in this structure, finally leading to reduction of PD motor deficits. Still, it has to be mentioned that elevated concentration of BDNF and following dopamine levels increase are not single factors, which restricts neural apoptosis in the substantia nigra in trained PD subject. ET also causes the reduction of chronic oxidative stress and strong activation of antioxidant enzymes [163], which powerfully decrease the risk of PD development.

## 4. Training-Evoked Increase in the BDNF Level Prevents Symptoms of the Metabolic Syndrome

Unbalanced diet and low physical activity are the main factors leading to the MetS, which is currently a most common metabolic disorder affecting from 10 to 50% of worldwide population [164, 165]. MetS is defined as a cooccurrence of such symptoms as obesity with many complications like hyperglycemia, proinflammatory state, dyslipidaemia, impaired glucose tolerance, hypertension, or cardiovascular and kidney diseases [166, 167]. Moreover, considerably reduction of vascular tissue of NGF and circulating NGF and BDNF levels was demonstrated by Chaldakov et al. [168] in a severe form of MetS and advanced coronary atherosclerosis in humans, which suggests that NGF and BDNF may be involved in the development of MetS, cardiovascular disease, and related disorders.

The adipose tissue is not only a main place of energy storage in organism but also active endocrine organs, which secretes a variety of bioactive molecules collectively termed as adipokines or adipocytokines [169, 170]. The most important peptide hormone produced by visceral adipose tissue is adiponectin [171], which contributes to the regulation of glucose [172] and fatty acid metabolism [173] as well as cardiovascular homeostasis [174]. Adiponectin influences, among others, on the levels of interleukin 6 (proinflammatory cytokine), tumor necrosis factor-alpha (regulator of insulin resistance), leptin (regulator of energy balance and suppressor of food intake), and angiotensinogen PAI-1 (regulator of blood pressure and fluid balance) [175]. It was demonstrated that altered concentration of adiponectin changes the glucose homeostasis, the insulin resistance, and the enhanced inflammatory processes [176, 177]. Therefore, maintaining the stable level of adiponectin is important in the prevention of metabolic disorders like obesity [178], diabetes type 2 [179], hypertension, and cardiovascular diseases [180, 181].

It is commonly known that regular physical activity is a treatment leading to limitation of MetS symptoms. For example, Cameron et al. [18] showed that ET can be successfully used to prevent the development of MetS. They demonstrated that rats fed with a high-carbohydrate high-fat diet (HCHF) (the model of human MetS) had elevated blood pressure, increased interstitial collagen in the left ventricle, mass of the liver, and higher activity of liver transaminases, which contributes to the accumulation of fat. Moreover, high concentrations of triglycerides, cholesterol, nonesterified fatty acids, and glucose and inefficiency of insulin responses were described as effects of HCHF feeding. In contrast to sedentary HCHF animals, trained HCHF rats had similar values of studied parameters to rats from either not trained or not feeding of HCHF groups.

It should be stressed that combination of endurance activity and caloric restriction is extremely effective in reduction of body weight, lowering the glucose and cholesterol level and normalization of systolic blood pressure [164, 182]. Recently, Aparicio et al. [183] investigated the influence of caloric restriction diet (30% reduced food intake) and interval aerobic training combined with strength-endurance exercise (IASE) in obese rats. It was noticed that trained rats with 30% reduced diet had a significantly lower level of total cholesterol, LDL, phospholipids, HOMA-IR (parameter for insulin resistance), adiponectin, inflammatory markers, and glucose than sedentary rats and 16% higher level of HDL. Moreover, concentration of triglycerides in plasma was reduced about 50% as compared to trained and untrained groups of animals with unlimited access to food.

Such spectacular effects of training-diet combination could be explained by a considerable increase of posttraining BDNF concentration in the blood observed in numerous experiments [102, 124]. Also, food limitation may cause an additional increase in the BDNF concentration, which accelerates the effects of ET. The results of the study performed by Araya et al. [184] that investigated the effects of reduced-calorie diet in obese subjects revealed the increase of the BDNF concentration in blood serum after three months of diet. Recently, Bastani et al. [185] indicated that caloric restriction (month of Ramadan) significantly increases the concentration of BDNF and NGF in human plasma. Therefore, elevation of the BDNF concentration in the bloodstream evoked by endurance activity, limited caloric intake, or their combinations seems to be a key for the prevention of various symptoms of MetS.

### 4.1. The Prevention of Obesity

The hypothalamic nuclei are important parts of the brain involved in the regulation of metabolism, due to their ability to synthesize and release various neuropeptides such as leptin, urocortin, and corticotrophin-releasing hormone, which influence feeding behavior [186]. Numerous hypothalamic nuclei contain TrkB-expressing neurons [99], which are capable to produce BDNF [68]. This suggests that BDNF can act directly on the hypothalamic environment responsible for the regulation of appetite and food intake. Kernie et al. [187] observed the development of eating disorder leading to obesity in mice with reduced BDNF gene expression and demonstrated that infusion of BDNF or NT4/5 to the brain can reverse this process. Such result directly supported the idea that TrkB signaling in the hypothalamic nuclei is responsible for the regulation of appetite and food intake. Also, Tsao et al. [188] indicated that peripheral administration of NT4 suppresses appetite and reduces body weight in obese mice. Moreover, these authors indicated that NT4 treatment evokes increased lipolysis, reduced body fat content and leptin, and elicits long-lasting amelioration of hypertriglyceridemia and hyperglycemia. Reduction of food intake was also observed after intraperitoneal administration of BDNF in two different models: obese mice kept on high-fat diet and genetically modified mice with leptin resistance [189]. Study of Toriya et al. [190] brought evidences that long-term infusion of BDNF via an osmotic minipump not only increases the BDNF concentration in the hypothalamus nuclei of mice but also inhibits food intake and increases energy expenditure due to upregulation of the mRNA expression of corticotrophin-releasing hormone and urocortin. Finally, Woo et al. [191] found that exercise training significantly increases the BDNF expression in the hypothalamus, reduces the leptin level in plasma, and causes weight loss in obese rats fed with the high-fat diet. These latter results directly indicated that high levels of BDNF in the hypothalamus evoked by training is effective for the prevention of obesity.

Results coming from animal experiments cited above suggest a great therapeutic potential of training-evoked BDNF in the treatment of obesity. Detailed mechanism of anorexic action of BDNF on hypothalamic nuclei is not fully understood. However, Takei et al. [99] considered that BDNF can act on hypothalamic nuclei-controlling metabolism by activation of mammalian target of rapamycin (mTOR) known as brain food intake regulator [192] which is a kinase that governs metabolism in peripheral cells.

### 4.2. The Prevention of Diabetes

Type 1 diabetes mellitus (T1D) is a chronic disease characterized by autoimmune destruction of beta cells in the pancreas, which is responsible for the synthesis and exertion of the insulin [193, 194]. This disease usually appears when 80–95% of beta cells are destroyed, which leads to hyperglycemia [195]. Deficiency of insulin and also significantly elevated levels of proinflammatory cytokines such as TNF-*α* and interleukin 6 (IL-6) are observed in this disease [196]. Increased concentrations of TNF-*α* and IL-6 are associated with the possibility of occurrence of atherosclerosis and cardiovascular diseases [197]. From this reason, T1D is often considered as risk factors for cardiovascular death due to myocardial infarction, stroke, etc. [198].

Some observations indicated that exercises can be a promising strategy against pathological changes evoked by T1D. For example, Tonoli et al. [199] observed a significant decrease of glucose and increase of BDNF levels in the blood of patients with T1D, after continuous and interval training. Recently, da Silva et al. [200] evaluated the effects of low-intensity swimming training on the level of cardiac cytokines, as well as structural and contractile properties of cardiomyocytes in diabetes rats induced by streptozotocin which exhibit typical symptoms of T1D. It was found that regular endurance exercise reduces the TNF-*α* and collagen fibers in the left ventricle, increased the concentration of adiponectin involved in the regulation of glucose levels and the level of anti-inflammatory interleukin-10, and increased the density of capillaries.

Type 2 diabetes (T2D) is the most common form of diabetes constituting about 90% of the diabetic population, characterized by elevated levels of plasma glucose which is caused by impairment in both insulin secretion and its action [201]. T2D is a typical lifestyle disease, which can be prevented by changes in dietary habits and by an increase of physical activity [202]. Krabbe et al. [203] demonstrated that impaired glucose metabolism is associated with low levels of BDNF in humans and suggested that circulating levels of BDNF are regulated in response to plasma levels of glucose. This leads to conclusion that decreased BDNF may be a pathogenic factor involved in T2D.

Recent report of Winding et al. [204] shows that regular high-intensity interval training and endurance exercises are effective in glycemic control of humans with T2D. High reduction of body weight and fat mass and decrease of postprandial glucose were observed after such types of training. Marcinko et al. [20], who studied the influence of high-intensity interval training on insulin sensitivity in obese mice, also demonstrated a decrease in glucose levels in the blood after such activity. Eslami et al. [205] showed that six weeks of endurance activity was enough either to considerably reduce blood glucose or to considerably increase the BDNF expression in diabetic rats. Taken together, these results confirmed that neurotrophic support is necessary to prevent diabetes because it ameliorates glucose balance and improves insulin sensitivity [206].

Bathina and Das [207] suggested the enhancement of the BDNF level due to its production in the brain and gut after exercises. Moreover, these authors proposed that gut-evoked BDNF can act on pancreatic *β* cells which not only improves their survival but also enhances proliferation [207]. Therefore, such mechanism for counteracting both types of diabetes seems to be very likely.

### 4.3. The Prevention of Hypertension

Hypertension is a progressive cardiovascular disease, in which the blood pressure in the arteries is persistently elevated [208]. Long-term high blood pressure is a major risk factor for many cardiac conditions like stroke, heart failure, and coronary artery disease [209]. Studies performed in humans have indicated that exercise training reduces blood pressure in patients with hypertension [210] and prevents the development of this syndrome in adults with normal blood pressure [211]. Moreover, in humans, the reduction of both systolic and diastolic blood pressure persists for some time especially after accumulated rather than continuous sessions of physical activity [212]. Data collected from animal experiments also support the idea that ET is a highly effective method in treating hypertension. For example, the study performed by Holloway et al. [213] showed that four weeks of classical, progressive endurance activity is sufficient to reduce the majority symptoms of hypertension evoked by high saline diet in rats. Wright et al. [22] demonstrated that long-term ET on treadmill can not only decrease body weight and body fat but also reduce cardiac fibrosis, which is known as a serious cardiological problem resulting from the accumulation of glycation-end products acting on collagen in the extracellular space of the heart in response to hypertension [214, 215].

It should be stressed that low levels of circulating BDNF were found in patients with cardiovascular disorders associated with hypertension. For example, reduced plasma or serum levels of BDNF were noted in patients with increased risk of stroke [216], with chronic heart failure [217] or with acute coronary syndromes [77]. Therefore, elevation of the BDNF level has been proposed as “the mechanism by which physical exercise reduces blood pressure and lowers hypertension risk” in humans [218]. Similar conclusion resulted from the study of Prigent-Tessier et al. [219], which demonstrated that treadmill training was highly effective in the upregulation of BDNF even in rats with high blood pressure (i.e., in spontaneously hypertensive rats). In addition, this study showed high posttraining BDNF concentration in the heart and aorta of rats, which expression was the most prominent in endothelial cells. Effects of endurance activity described in this report were concluded that “physical training and hypertension have opposite effects on endothelial BDNF expression.” Therefore, consistent results coming from numerous reports involving humans and animals confirmed the beneficial effects of BDNF in the regulation of blood pressure.

## 5. Conclusion

**Figure 1:**
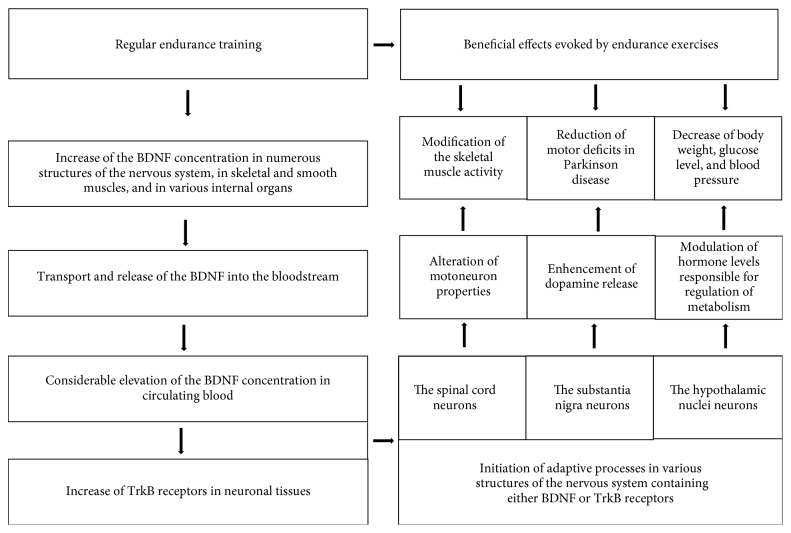
Scheme of pleiotropic action of training-evoked BDNF on neurons located in the spinal cord, in the substantia nigra, and in the hypothalamic nuclei, which control the motor performance and metabolic function of the body.

This review presents only a part of broad-spectrum effects that ET exerts on several aspects of mammal physiology. However, even presented data, which are limited to several aspects in this paper, show that (1) low concentration of endogenous BDNF in the blood indicates a high risk of neurodegenerative or metabolic diseases, (2) long-time regular ET and restriction of caloric intake elevate the endogenous concentration of BDNF in the blood and the expression of TrkB receptors in neurons, (3) a significant increase of BDNF in circulating blood appears mainly after aerobic exercise, which can be considered as proper strategy for BDNF enhancement in circulating blood, than after different types of physical exercises (e.g., strength training), (4) the largest amount of BDNF is produced by the brain structures, while synthesis and secretion of BDNF from nonneuronal sources (the skeletal muscles, gut) is rather limited to the local environment, (5) an increase of training-evoked BDNF in the bloodstream triggers pleiotropic, adaptive changes in neuronal and nonneuronal tissues, organs, and structures with TrkB or p75 receptors (Figure 1), (6) there is no single common mechanism of adaptation evoked by BDNF, due to its contribution to many different biochemical processes, and finally, (7) details of many adaptive changes evoked by BDNF are not fully recognized at the molecular level.
